# CRP2, a new invadopodia actin bundling factor critically promotes breast cancer cell invasion and metastasis

**DOI:** 10.18632/oncotarget.7327

**Published:** 2016-02-11

**Authors:** Céline Hoffmann, Xianqing Mao, Monika Dieterle, Flora Moreau, Antoun Al Absi, André Steinmetz, Anaïs Oudin, Guy Berchem, Bassam Janji, Clément Thomas

**Affiliations:** ^1^ Laboratory of Experimental Cancer Research, Department of Oncology, Luxembourg Institute of Health, 84 Val, Fleuri, Luxembourg, Luxembourg; ^2^ NorLux Neuro-Oncology Laboratory, Department of Oncology, Luxembourg Institute of Health, 84 Val, Fleuri, Luxembourg, Luxembourg; ^3^ Department of Oncology, Luxembourg Institute of Health, 84 Val, Fleuri, Luxembourg, Luxembourg

**Keywords:** actin cytoskeleton, breast cancer, invadopodia, invasion, LIM domain, MMP-9

## Abstract

A critical process underlying cancer metastasis is the acquisition by tumor cells of an invasive phenotype. At the subcellular level, invasion is facilitated by actin-rich protrusions termed invadopodia, which direct extracellular matrix (ECM) degradation. Here, we report the identification of a new cytoskeletal component of breast cancer cell invadopodia, namely cysteine-rich protein 2 (CRP2). We found that CRP2 was not or only weakly expressed in epithelial breast cancer cells whereas it was up-regulated in mesenchymal/invasive breast cancer cells. In addition, high expression of the CRP2 encoding gene *CSRP2* was associated with significantly increased risk of metastasis in basal-like breast cancer patients. CRP2 knockdown significantly reduced the invasive potential of aggressive breast cancer cells, whereas it did not impair 2D cell migration. In keeping with this, CRP2-depleted breast cancer cells exhibited a reduced capacity to promote ECM degradation, and to secrete and express MMP-9, a matrix metalloproteinase repeatedly associated with cancer progression and metastasis. In turn, ectopic expression of CRP2 in weakly invasive cells was sufficient to stimulate cell invasion. Both GFP-fused and endogenous CRP2 localized to the extended actin core of invadopodia, a structure primarily made of actin bundles. Purified recombinant CRP2 autonomously crosslinked actin filaments into thick bundles, suggesting that CRP2 contributes to the formation/maintenance of the actin core. Finally, CRP2 depletion significantly reduced the incidence of lung metastatic lesions in two xenograft mouse models of breast cancer. Collectively, our data identify CRP2 as a new cytoskeletal component of invadopodia that critically promotes breast cancer cell invasion and metastasis.

## INTRODUCTION

Metastasis is the primary cause of death from cancer and is a major hurdle for cancer treatment [1]. A critical step of the metastatic cascade is the acquisition by carcinoma cells of the ability to remodel the extracellular matrix (ECM) and migrate through the stromal environment and tissue barriers. At the subcellular level, such ability is associated with actin-rich membrane protrusions termed invadopodia, which recruit and drive the local secretion of matrix metalloproteinases (MMPs) able to promote ECM degradation [2, 3]. Invadopodia were initially described on the basal surface of cultured cancer and v-Src transformed cells in the 80's [4]. However, their relevance in cancer progression and potential as therapeutic targets have only recently been recognized [5-7]. For instance, a recent intravital imaging-based study has provided direct and compelling evidence of the key role played by invadopodia during the extravasation of human breast cancer cells in mouse models [6]. Although initially promising, MMP inhibitors failed in the clinic, mostly due to high toxicity [8], and new molecular targets to inhibit invadopodia formation and/or activity are required.

Invadopodia comprise a protrusive actin-dense core and a surrounding region enriched in signaling and adhesion proteins [3, 9]. In the core, actin filaments (AFs) are cross-linked in thick bundles, which presumably focus actin polymerization-promoted force for protrusive activity, and stabilize invadopodia over long periods to optimize ECM degradation [9-12]. Consistent with such important roles for actin core bundles, the invasive and metastatic potential of tumor cells was considerably reduced by inhibiting the expression or activity of fascin, an invadopodia-enriched actin-bundling factor [9, 10, 12-14]. Although T-fimbrin, another AF crosslinking protein, was also detected in invadopodia, its knockdown only had minimal effects on invadopodia biogenesis and activity [9], suggesting that fascin is the major actin bundling factor in invadopodia.

Cysteine-rich proteins (CRPs) define an evolutionary-conserved subfamily of short (21 kDa) LIM domain proteins characterized by two LIM domains [15, 16]. The three vertebrates CRPs (CRP1-3) exhibit a dual cytoplasmic and nuclear localization, and are preferentially expressed in muscle tissues [17]. In the nucleus, CRPs can interact with transcription factors, such as MyoD, SRF and GATA family proteins, to facilitate smooth (CRP1 and CRP2) or striated muscle (CRP3) differentiation [18, 19]. In the cytoplasm, they decorate filamentous actin structures and bind to cytoskeletal proteins, such as α-actinin and zyxin [17]. However, their exact functions in this compartment remain unclear. Missense mutations in human CRP3 have been associated with dilated and hypertrophic cardiomyopathy [20-22], and CRP3 ablation in transgenic mice promotes disruption of cardiac cytoarchitecture organization, cardiomyopathy and heart failure [23, 24]. We recently provided evidence that CRP3 binds to filamentous actin and self-associates to assemble AFs into bundles, suggesting that it has a direct structural role in cytoarchitecture maintenance [25]. Others reported that CRP1 localizes to neuron filopodia and critically regulates filopodia formation and dendritic growth by a mechanism involving AF crosslinking [26, 27]. Here, we show that CRP2 is an autonomous actin bundling protein whose expression is up-regulated in highly invasive breast cancer cells, and that accumulates along and within the actin core of mature invadopodia. In addition, we provide evidence that this previously overlooked cytoskeletal component of invadopodia promotes breast cancer cell invasiveness and metastasis, and we discuss its potential as a therapeutic target.

## RESULTS

### CRP2 is up-regulated in aggressive breast cancer tumors and cell lines

**Figure 1:**
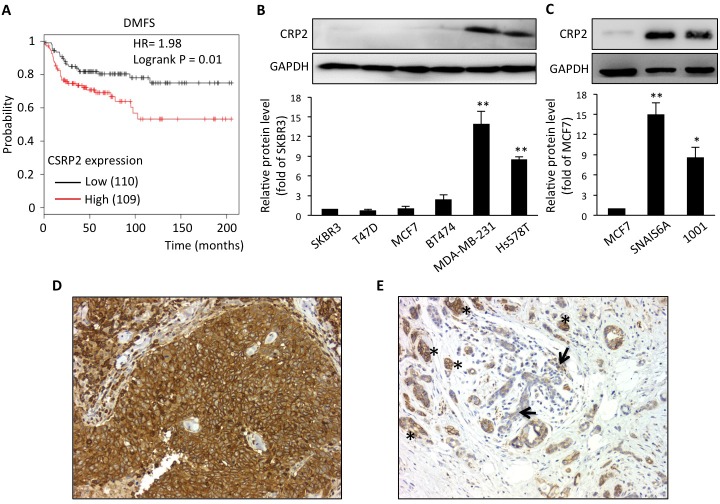
CRP2 up-regulation is associated with a significantly higher risk of metastasis in basal-like breast cancer patients, and correlates with the mesenchymal phenotype in human breast cancer cell lines **A.** Kaplan-Meier survival analyses in relation to *CSRP2* expression (affy ID 207030_s_at) in breast carcinoma from the basal subtype using distant metastasis free survival as an endpoint. The patient samples, hazard ratio with 95% confidence interval, and *p* value (Logrank test) are displayed on the chart. **B.** and **C.** CRP2 protein level in human breast cancer cell lines (B) and in MCF7-derived cells which underwent EMT through the expression of a constitutively active version of Snail (SNAIL S6A) or prolonged TNF treatment (1001; C). Relative CRP2 expression (lower panels) are calculated from at least three independent experiments and expressed as fold of CRP2 protein level in SKBR3 (B) or MCF7 cells (C). **D.** and **E.** Immunohistochemical staining of CRP2 in two cases of invasive ductal carcinoma showing strong staining in tumor cells (D), and faint staining in residual normal breast tissue (arrows; strongly stained tumor cells are indicated by asterisks; (E), respectively (magnification: 200x). Error bars denote standard error. Significant levels: *: *p* < 0.05 and **: *p* < 0.001 (unpaired, two-tailed *t*-test).

A microarray-based analysis identified the CRP2 encoding gene, *CSRP2*, in a cluster of 14 genes whose high expression is characteristic of basal-like breast carcinoma [28], a breast cancer subtype associated with poor prognosis. To further evaluate the prognostic value of *CSRP2*, we conducted *in silico* survival analyses using publicly available gene expression datasets with well-defined patient clinical follow-up [29]. Kaplan-Meier and logrank tests revealed that, within the basal-like subtype, breast cancer patients with higher expression of *CSRP2* in the primary tumor exhibit significantly reduced metastasis-free survival as compared to patients with lower expression of *CSRP2* (Figure 1A; HR = 1.98, *P* = 0.01). In parallel, immunohistochemical analyses revealed that of the 48 invasive breast cancer cases analyzed, 17 (35.4%) were negative, 27 (56.3%) showed weak to moderate staining and 4 (8.3%) exhibited strong staining (Figure 1D). Some residual normal cells were also labeled but they usually exhibited weaker staining as compared to the tumor cells in the same tissue sample (Figure 1E). Interestingly, inflammatory cells also exhibited strong staining (data not shown). To assess the possibility that CRP2 expression was associated with the intrinsic invasive/metastatic potential of breast tumor cells, we compared CRP2 protein levels in a range of well-characterized human cell lines. As shown in Figure 1B, only low levels of CRP2 protein were detected in non- or poorly metastatic, epithelial, breast cancer cells [30], including SKBR3, T-47D, MCF-7 and BT474 cells. In contrast, CRP2 protein levels were considerably higher in highly metastatic, mesenchymal, breast cancer cells, including MDA-MB-231 and Hs548T cells. We analyzed two additional cell lines derived from MCF-7 cells that underwent an epithelial-to-mesenchymal transition (EMT) following either prolonged TNF treatment (1001 cells [31]), or expression of constitutively active Snail (SNAI1-S6A cells [32]). As shown in Figure 1C, both 1001 and SNAI1-S6A cells exhibited a substantial increase in CRP2 protein as compared to the parental epithelial MCF-7 cells. Together the above data suggest that CRP2 up-regulation is associated with the mesenchymal/invasive breast cancer cell phenotype and an increased risk of metastasis in patients.

### CRP2 localizes to breast cancer cell invadopodia

**Figure 2:**
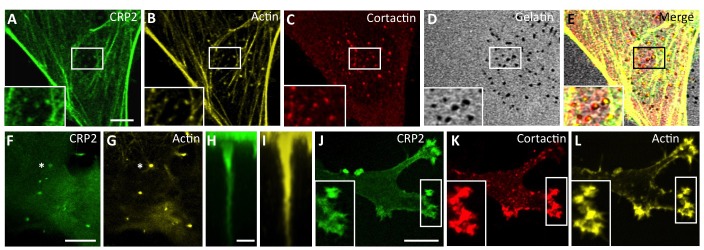
CRP2 localizes to active invadopodia **A**.-**D**. CRP2-GFP localizes to actively degrading invadopodia. Cells were grown on Cy3-conjugated gelatin-coated coverslips (D) for 6 h and CRP2-GFP A. was co-localized with actin (B) and cortactin (C). **E**. Merge of (A-D) **F**.-**I**. CRP2-GFP (F) and (H) and actin (G) and (I) co-localize in elongated invadopodia. F. and G. correspond to a focal plane located at 1.4 μm underneath the ventral cell surface, whereas (F) and I show projections along the z axis (35 confocal slices) of the entire invadopodium labeled by an asterisk in (F) and (G) **J**.- **L**. CRP2-GFP (J), cortactin (K) and actin (L) in MDA-MB-231-luc cells invading a 3D EHS matrix. The image corresponds to a projection of 17 confocal slices. Bars = 5 μm (A-G), 1 μm (H) and (I), 10 μm (J-L).

To get an insight into the role of CRP2 in breast cancer cells, CRP2 was fused to GFP and expressed in highly metastatic, Basal-like, MDA-MB-231-luc-D3H2LN cells [33] (hereafter referred to as MDA-MB-231-luc cells). As shown in Figure 2A-2E, CRP2-GFP extensively decorated actin stress fibers. In addition, it co-localized with actin (Figure 2B) and cortactin (Figure 2C), a critical invadopodia protein [34], in invadopodia actively engaged in matrix degradation (Figure 2D). In contrast with other LIM proteins, such as paxillin and Hic-5, which accumulate in a ring surrounding punctate degraded areas [35], CRP2-GFP localized in the invadopodia core overlying the areas of gelatin degradation (Figure 2E). To refine CRP2 localization, we used a chemoinvasion assay in which invadopodia can elongate over long distances through 1-μm-diameter pores in response to growth factors [9]. Projection along the z-axis of confocal sections revealed that CRP2-GFP decorates the whole length of extended actin cores (Figure 2F-2I, and Supplementary Movie 1). Noticeably, such a distribution was highly similar to that previously reported for fascin [9], suggesting functional interaction or redundancy between both CRP2 and fascin in invadopodia. To extend our analysis, the endogenous CRP2 protein was detected in MDA-MB-231-luc cells, as well as in another invasive breast cancer cell line, namely Hs578T. According to the above GFP fusion data, endogenous CRP2 accumulated within the invadopodia actin core in both cell types (Supplementary Figure 1 and Supplementary Figure 2A, respectively). Finally, CRP2-GFP was localized in MDA-MB-231-luc cells embedded in a 3D matrix [36, 37], a condition that better mimics the tumor microenvironment. It was previously reported that, in 3D Matrigel, MDA-MB-231-luc cells adopt an elongated shape, develop cell-matrix adhesions [38] and invade by reshaping the surrounding matrix in a protease dependent manner [37]. As shown in Figure 2J-2L, CRP2-GFP accumulated in actin- and cortactin-enriched areas located at the cell leading edge, in extending pseudopodia, supporting the idea that CRP2 contributes to the mesenchymal mode of invasion in breast cancer cells.

### CRP2 is an autonomous actin-bundling protein

**Figure 3:**
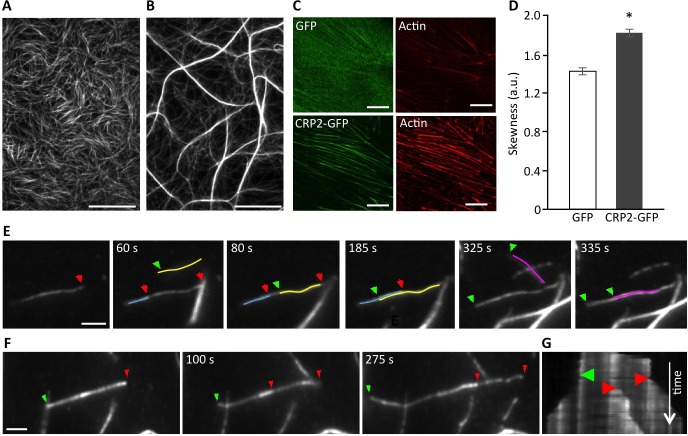
CRP2 promotes actin bundling in *in vitro* reconstituted assays and in breast cancer cells **A.** and **B.** Actin filaments (1 μM) polymerized alone (A) or in the presence of recombinant CRP2 (3 μM; B). **C.** Typical examples of ROI (13 × 13 μm) used for skewness measurements in Acti-stain 555 phalloidin-stained MDA-MB-231-luc cells expressing GFP alone or CRP2-GFP. **D.** Skewness average calculated from three independent experiments, including 200 optical sections as in (C). Error bars denote standard error. Significant level: *: *p* < 0.001. **E.** and **F.** Selection of images from Supplementary Movies 2 and 3 (real-time time TIRF microscopy) showing CRP2-induced crosslinking of actin filaments (E) and actin filaments elongation inside a bundle (E) and (F). In both cases 1 μM actin was copolymerized with 3 μM CRP2. Green and red arrows point to fast growing barbed ends of filaments elongating toward the left and right, respectively. For better readability some actin filaments were highlighted in color. G. Kymograph corresponding to (F) and Supplementary Movie 3, showing that CRP2 assembles bundles of mixed polarity. Bars = 20 μm (A and B), 10 μm C., 2 μm (E and F).

Recently, both CRP1 and CRP3, as well as several plant CRP-like proteins, were reported to regulate actin cytoskeleton organization and dynamics [16, 25, 26, 39-41]. The importance of actin bundling for invadopodia biogenesis and activity (9) prompted us to evaluate the ability of CPR2 to remodel AF networks. To do so, AFs were co-polymerized with recombinant CRP2, and the resulting structures were analyzed by real-time total internal reflection fluorescence (TIRF) microscopy. In a control experiment conducted in the absence of CRP2, actin polymerization generated a randomly organized meshwork of fine AFs (Figure 3A). In contrast, in the presence of CRP2, AFs organized in a reticulated network of thick and long actin bundles (Figure 3B). The formation and growth of CRP2-promoted bundles resulted from the fusion of preexisting AFs or fine bundles, and elongation of individual AFs within bundles (Figure 3E and 3F, respectively; Supplementary Movies 2 and 3). By tracking the fast-growing end of bundled AFs, we established that CRP2 has weak intrinsic selectivity for AF polarity and mostly assembles actin bundles of mixed polarity (Figure 3E, 3F and 3G). To extend these data, we analyzed the cytoskeletal modifications potentially induced by CRP2 overexpression in MDA-MB-231 cells. For practical reasons, we focused on actin stress fibers as these structures are easily amenable to quantitative analyses. As shown in Figure 3C, the stress fibers of CRP2-GFP expressing cells appeared considerably thicker than those of control cells expressing GFP alone. The extent of actin bundling in both cell lines was quantified as previously described in Hoffmann *et al.* [25]. From three independent experiments, including about 200 optical sections, we calculated an average skewness value (a reliable indicator of actin bundling) of 1.43 ± 0.04 and 1.83 ± 0.04 for GFP control cells and CRP2-GFP expressing cells, respectively (Figure 3D). In conclusion, our data demonstrate that CRP2 exhibits actin bundling activity in both *in vitro* reconstitution assays and breast cancer cells.

### CRP2 critically regulates breast cancer cell invasion

**Figure 4:**
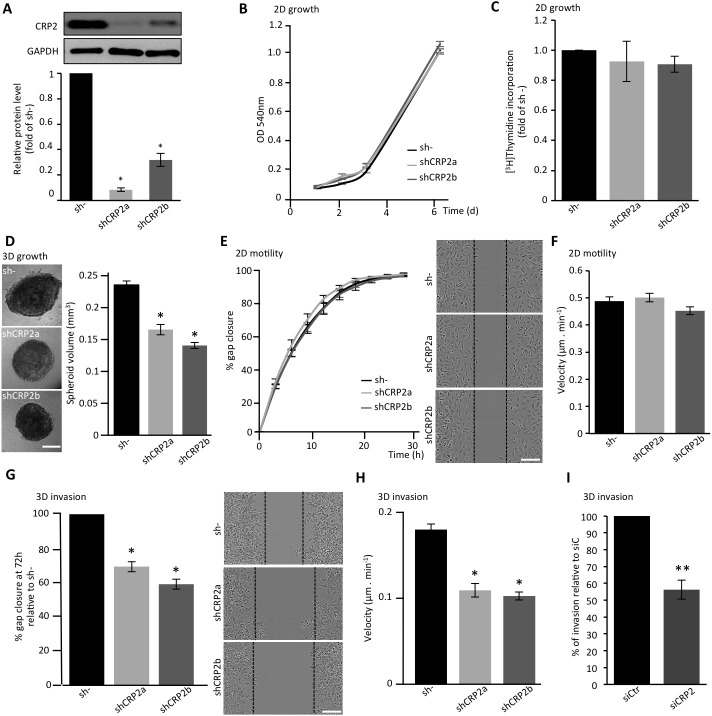
CRP2 contributes to breast cancer cell invasion **A.** CRP2 protein level in MDA-MB-231-luc cell lines stably expressing two independent shRNAs targeting CRP2 transcripts (shCRP2a and shCRP2b) or a control, non-targeting, shRNA (sh-). Average expression values (lower panel) in shCRP2a and b cell lines were calculated from three independent western blot analyses and expressed as fold of CRP2 protein in sh- cells. **B.** Proliferation rate of sh-, and shCRP2a and b cell lines as assessed by MTT assay. **C.** Proliferation rate as assessed by [^3^H]Thymidine incorporation. The data are expressed as fold of [^3^H]Thymidine incorporation in control sh- cells. Unpaired two-tailed, *t*-test revealed no statistical difference in the proliferation rate of the three cell lines. **D.** 3D cell spheroid proliferation assay. The right chart indicate the average spheroid volume at 6 days of culture (*n* = 25). **E.** 2D scratch wound assay (on 2D collagen matrix surface). Gap closure was analyzed using the automated image acquisition and processing system IncuCyte (Essen BioScience). The right image panel show typical gap closure at 6h for each cell line. **F.** Velocity of 2D migrating cells. For each cell line, at least 150 cells migrating on collagen-coated μ-Slide Chemotaxis^2D^ chambers (Ibidi) were tracked over 2 days, and an average velocity was calculated. **G.** 3D scratch wound assay. After wounding, cells were embedded in a 3D collagen matrix. Results were normalized to sh- and are expressed as percentage of gap closure after 72h. The right image panel show typical gap closure at 72 h for each cell line. **H.** Velocity of invading cells. For each cell line, at least 150 cells embedded in a 3D collagen matrix were tracked over 2 days and an average velocity was calculated. **I.** Transwell assay performed with MDA-MB-231-luc cells transfected with control (siCtr) or CRP2 specific (siCRP2) siRNA. Invading cells at 24 h were quantified by MTT assay, and the results were normalized to siCtr cells (set at 100%). All the data originate from at least three independent experiments. Error bars denote standard error. Significant levels: *: *p* < 0.001, **: *p* < 0.05. Bars = 300 μm (D) and 150 μm (E and G).

To further characterize the function of CRP2 in breast cancer cells, a series of *in vitro* functional assays were conducted with two MDA-MB-231-luc-derived cell lines expressing independent CRP2-targeting shRNAs (shCRP2a and shCRP2b), and one control cell line expressing a non-targeting control shRNA (sh-; Figure 4A). Both shCRP2a and shCRP2b cells exhibited significantly reduced CRP2 protein levels as compared to sh- cells (Figure 4A), and were checked for the absence of any CRP1 or CRP3 up-regulation, which could compensate for CRP2 knockdown (Supplementary Figure 3). Both MTT and [^3^H]Thymidine incorporation assays revealed that CRP2 knockdown did not affect the proliferation rate of MDA-MB-231-luc cell monolayer cultures (Figure 4B and 4C, respectively). Three-dimensional (3D) spheroids were successfully obtained from both control (sh-) and CRP2-depleted cells, and expanded as compact spheres in 10% Engelbreth-Holm-Swarm (EHS) matrix (Figure 4D). However, CRP2 knockdown significantly reduced 3D spheroid growth. Indeed, after 6 days of culture, sh- spheroids exhibited an average volume of 0.237 ± 0.006 mm^3^ whereas shCRP2a and shCRP2b only reached 0.167 ± 0.008 and 0.141 ± 0.005 mm^3^, respectively (Figure 4D, right chart). The migratory potential of each cell line was then evaluated in both 2D and 3D conditions. In 2D scratch wound assays, nearly identical closure curves were obtained for sh-, shCRP2a and shCRP2b cells, indicating that CRP2 depletion does not alter 2D cell migration (Figure 4E). This result was confirmed by the similar average migration velocities (~ 0.5 μm/min^−1^) that were calculated for each cell line by tracking single cells migrating towards FBS in collagen-coated μ-Slide Chemotaxis^2D^ chambers (Ibidi; Figure 4F). In contrast, 3D scratch wound assays revealed that CRP2 knockdown significantly reduces the ability of MDA-MB-231-luc cells to invade into a 3D collagen matrix (Figure 4G). In these conditions, gap closure after 72h for shCRP2a and shCRP2b cells was reduced by about 30% and 40% as compared to control sh- cells, respectively. In addition, single cell tracking analyses showed that CRP2 depletion reduces by about 50% the average velocity of collagen invading cells (Figure 4H). To strengthen these data, transwell invasion assays were performed with MDA-MB-231-luc cells treated with control or CRP2 siRNA. As shown in Figure 4I, transient CRP2 knockdown inhibited the invasion potential of MDA-MB-231-luc cells by about 50%. Finally, the pro-invasive function of CRP2 was validated in Hs578T cells (Supplementary Figure 2B-2D). Indeed, both transwell invasion and single cell tracking analyses showed that CRP2 depletion significantly reduced Hs578T cell invasion.

**Figure 5:**
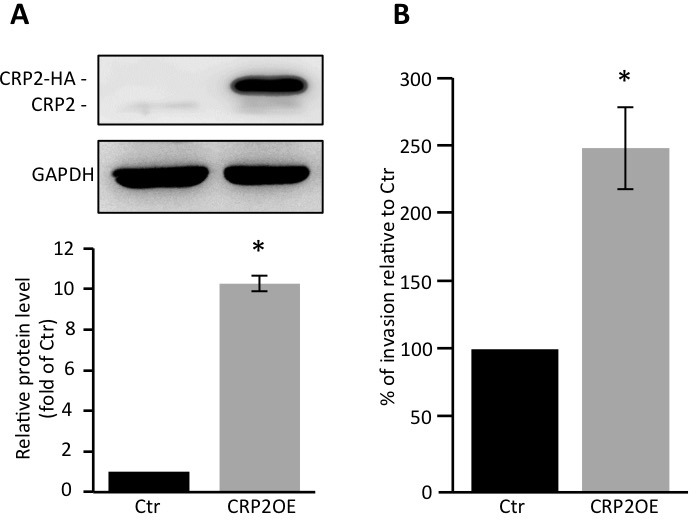
CRP2 overexpression promotes MCF-7 cell invasion **A.** CRP2 protein level in MCF-7 cell lines stably expressing CRP2-HA (CRP2OE) or a control empty vector (Ctr). Average total CRP2 expression (lower panel) are calculated from three independent western blot analyses and are expressed as fold of endogenous CRP2 protein in Ctr cells. **B.** Transwell invasion assay. Invading cells at 48 h were quantified by MTT assay, and the results were normalized to Ctr cells (set at 100%). The data originate from 5 independent experiments. Error bars denote standard error. Significant levels: *: *p* < 0.005.

The possibility that CRP2 up-regulation can promote breast cancer cell invasion in weakly invasive cells was tested in gain-of-function analyses using MCF-7 epithelial cells, which only contain very low levels of CRP2 protein (Figure 1B and 1C). We established a stable MFC-7 cell line in which CRP2 protein expression was about 10 times higher than in control MCF-7 cells (Figure 5A). Remarkably, CRP2 overexpression increased MCF-7 cell invasion by a factor 2.5 in transwell invasion assays (Figure 5B). Together our data provide clear evidence that CRP2 critically regulates breast cancer cell invasion whereas it is not required for 2D cell migration.

### CRP2 knockdown inhibits extracellular matrix degradation and MMP-9 expression

**Figure 6:**
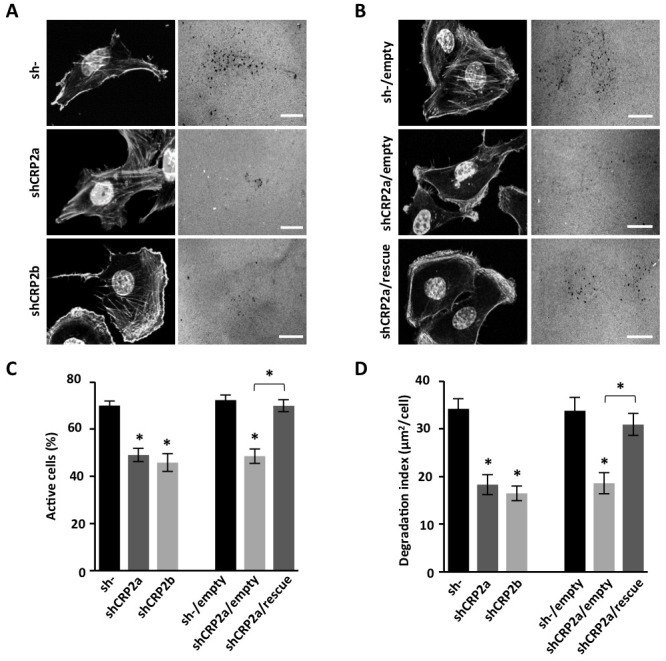
CRP2 is required for ECM degradation **A.** Gelatin degradation assay. Control sh- and shCRP2a and shCRP2b cells were plated on Cy3-conjugated gelatin for 18 h, fixed and stained with DAPI and Acti-stain^TM^ 670 phalloidin (left panels). Gelatin degraded areas appear as dark punctuate in the fluorescent background (right panels). **B.** Same as A for an shCRP2a-derived cell line in which CRP2 expression was restored by expressing an shRNA-resistant CRP2 coding sequence (shCRP2a/rescue), and related controls, i.e. sh- and shCRP2a cells lines transduced with an empty vector (sh-/empty and shCRP2a/empty, respectively; see also Supplementary Figure 4). **C.** and **D.** Quantitative analyses corresponding to the experiments shown in (A) and (B) Actively ECM degrading cells were scored and expressed as percentage of the total cell population (C). A degradation index corresponding to the ratio of degraded gelatin surface per cell was calculated (D). The data originate from at least three independent experiments (n ≥70 cells). Error bars denote standard error. Significant levels: *: *p* < 0.0001. Bars = 20 μm.

A hallmark of invading mesenchymal cells is their capacity to remodel/degrade the ECM and clear paths through challenging physical barriers, such epithelial and vascular basement membranes. Here, we assessed whether CRP2 knockdown can alter the ability of MDA-MB-231-luc tumor cells to promote ECM degradation. As shown in Figure 6A, control sh- MDA-MB-231-luc cells efficiently promoted fluorescent gelatin degradation as indicated by dark spots in the bright fluorescent matrix background. About 70% of the cell population was active and a degradation index of 34.2 ± 2.2 μm^2^ of degraded matrix per cell was calculated (Figure 6C and 6D, respectively). The depletion of CRP2 significantly reduced both parameters with only 49% and 46% of active cells and degradation indexes of 18.3 ± 2.1 and 16.5 ± 1.5 μm^2^/cell for shCRP2a and shCRP2b, respectively (Figure 6A, 6C and 6D). As shown in Figure 6B-6D, the rescue of CRP2 expression in shCRP2a cells (Supplementary Figure 4) restored matrix degrading activity to a similar level as in control cells, further confirming that the above effects were due to CRP2 knockdown.

**Figure 7:**
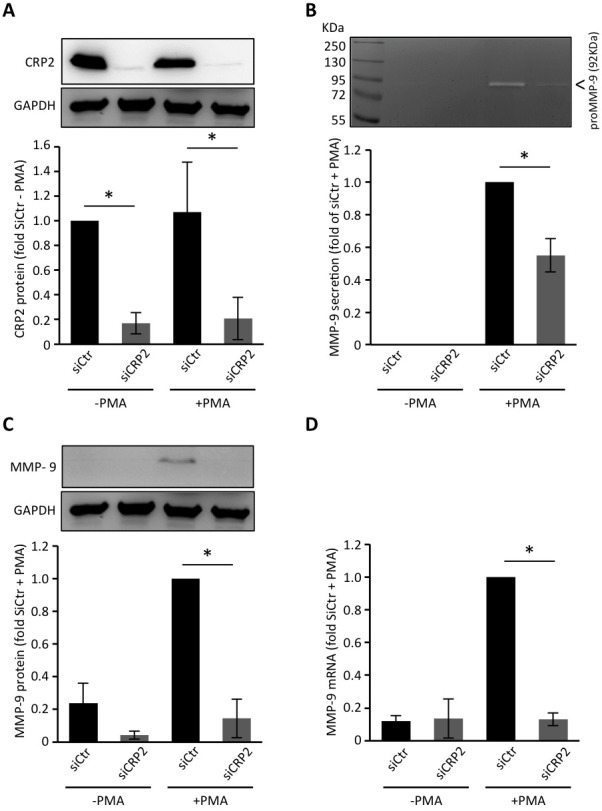
CRP2 regulates MMP-9 expression in MDA-MB-231-luc cells **A.** CRP2 protein level in MDA-MB-231-luc cells transfected with a control siRNA (siCtr) or a CRP2 targeting siRNA (siCRP2). Forty hours after transfection, cells weretreated with or without PMA (100 ng/ml) in serum-free DMEM for 24 h. Average expression values (lower panel) were expressed as fold of CRP2 protein in untreated (−PMA) control siCtr cells which was set to 1. **B.** Gelatin zymography assays. The medium of the cell cultures described in A. was collected and assessed for its content in secreted pro-MMP-9. The results were expressed as fold of pro-MMP-9 secretion in PMA-treated control siCtr cells which was set to1. **C.** and **D.** Expression levels of cytosolic MMP-9 protein (C) and MMP-9 mRNA (D) in the cell cultures described in A. as assessed by western blot and real-time qPCR analyses, respectively. The results were expressed as fold of pro-MMP-9 expression in PMA-treated control siCtr cells which was set to 1. The data originate from at least 3 independent experiments. Error bars denote standard error. Significant levels: *: *p* < 0.01.

Extracellular matrix degradation is primarily mediated by metalloproteinases (MMPs) that are secreted at sites of invadopodia. We thus aimed at evaluating the contribution of CRP2 to MMP secretion in MDA-MB-231-luc cells. Among secreted MMPs, the gelatinases MMP-2 and MMP-9 are of particular interest as they can hydrolyze major ECM components, such as gelatin and type IV collagen [42], and were repeatedly associated with breast cancer progression [43-45]. Previous studies established that MDA-MB-231 secrete low basal levels of MMP-2 and MMP-9 (i.e. when cultured in serum free media) and respond to phorbol 12-myristate 13-acetate (PMA) by secreting MMP-9 [46]. Using gelatin zymography analyses, we confirmed that MDA-MB-231-luc cells secrete no or very low basal levels of MMP-9 and MMP-2, and this was not modified by knocking down CRP2 expression (Figure 7A and 7B). As expected, PMA induced MMP-9 secretion (under its 92 kDa pro-form). Remarkably, CRP2 knockdown significantly compromised MMP-9 secretion. Expression analyses revealed that PMA induced MMP-9 expression at both protein and mRNA levels (Figure 7C and 7D), and that this process was abrogated in CRP2-depleted cells. Thus, the reduced MMP-9 secretion observed in CRP2 depleted cells was mostly due to the inhibition of MMP-9 expression. As shown in Supplementary Figure 5, these data were validated in Hs578T breast cancer cells, demonstrating that the role of CRP2 in MMP-9 expression/secretion is not restricted to the MDA-MB-231 cell lineage.

### CRP2 knockdown inhibits metastatic colonization

**Figure 8:**
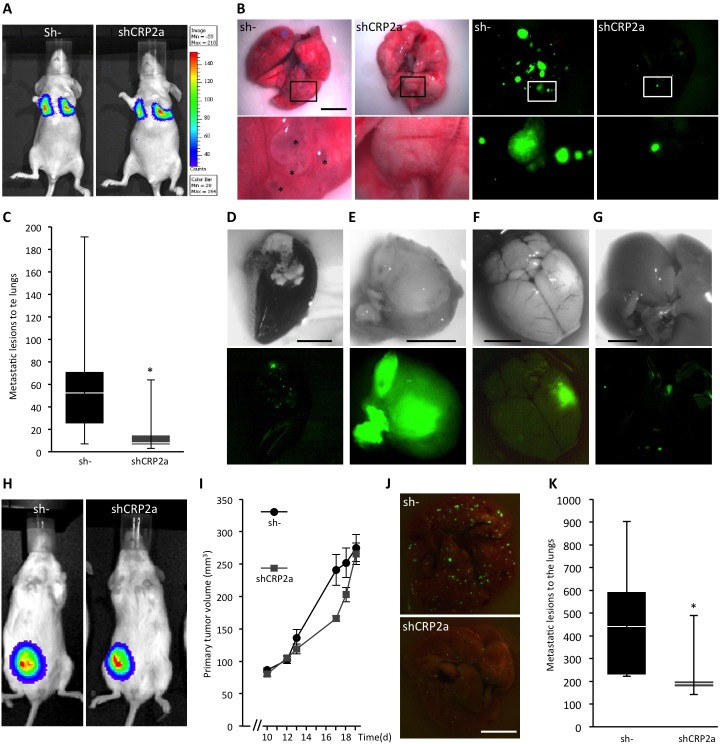
CRP2 promotes breast cancer metastasis **A.** Implantation of control (sh-) and CRP2-depleted (shCRP2a) MDA-MB-231-Luc cells (1×10^6^) to the lungs of athymic nu/nu female mice was immediately controlled by bioluminescence imaging after tail vein injection. **B.** Typical lung metastatic lesions observed after 5 weeks with white light (four left panels) and fluorescence microscopy to detect GFP (cell transduction marker; four right panels). Lower panels are magnifications corresponding to the above white rectangles. Asterisks indicate some extended metastatic lesions. **C.** Number of lung metastatic lesions in sh- and shCRP2a xenograft mice (*n* = 8 and 9, respectively). Data are presented as standard box plots with whiskers from minimum to maximum values. **D.** Extensively necrotized lung of one sh- mouse. **E.**-**G.** Metastases at other sites than lungs observed in sh- mice, including a hind leg (E; the image corresponds to the excised tumor), the brain (F) and the liver (G). The lower panels show the corresponding GFP (cell transduction marker) signals. **H.**-**K.** Orthotopic tumor model. Control (sh-) and CRP2-depleted (shCRP2a) MDA-MB-231-Luc cells (5×10^5^) were implanted in a mammary fat pad of NOD *scid* gamma mice (*n* = 5 and 6, respectively). **H**. Bioluminescence imaging showing typical and similar signals at the primary tumor site in sh- and shCRP2a xenograft mice (19 days after tumor cell implantation). **I.** Primary tumor volume. **J.** and **K.** Typical lung metastases (J) and number of lung metastatic lesions (K) 20 days after tumor cell implantation. Error bars denote standard error. Significant levels: *: *p* < 0.05. Bars = 5 mm.

Based on our data, we predict that CRP2 should significantly contribute to breast cancer metastasis. To test this assumption, CRP2-depleted (shCRP2a) and control (sh-) MDA-MB-231-luc cells (Figure 4A) were injected into the tail vein of athymic nu/nu female mice. Lung implantation of tumor cells was immediately controlled after tail vein injection by bioluminescence imaging (Figure 8A). After 5 weeks, lungs were excised and the number of GFP positive metastatic colonies was analyzed. As illustrated in Figure 8B, lesions were dramatically reduced in lungs from mice injected with CRP2-depleted tumor cells as compared to lungs from control mice. Quantitative analyses revealed that the median number of metastatic colonies was 8 and 53 for shCRP2a lungs and sh- lungs, respectively (Figure 8C; *p* < 0.05). Moreover, out of a total of 8 mice injected with sh- cells, one mouse exhibited extensively necrotized lungs (Figure 8D) and three developed macro-metastases at other sites than lungs, including hind legs, the brain and the liver, respectively (Figure 8E-8G). In striking contrast, none of the 9 mice injected with shCRP2a cells exhibited any sign of metastatic lesions in other tissues than lungs.

Since the above experimental metastasis model based on tumor cell intravascular injection does not recapitulate the early steps of the metastatic cascade, e.g. extravasation, we further examined CRP2 pro-metastatic activity in a spontaneous metastasis model. Here, CRP2-depleted and control MDA-MB-231-luc cells were injected in the mammary fat pad of NOD *scid* gamma (NSG) mice. The size of primary tumors was measured using calipers. As shown in Figure 8H and 8I, CRP2 knockdown had no significant effect on primary tumor growth. In contrast, the number of metastatic lesions to the lungs was significantly reduced (> 50%) in CRP2-depleted tumor bearing mice as compared to control mice (Figure 8J and 8K). These data are in good agreement with the established pro-invasive role of CRP2 and confirm that CRP2 promotes breast cancer metastasis.

## DISCUSSION

The present study provides evidence that CRP2 is a new actin bundling factor that localizes in breast cancer cell invadopodia and plays a critical role in invadopodia-mediated ECM degradation, cell invasion and metastasis. Although this is the first report of the presence of CRP2 in invadopodia, a proteomic study has recently identified CRP1 (which shares about 80% amino acid identity with CRP2) as a new component of podosomes [47], which are invadopodia-like organelles that promote ECM degradation in normal motile cells [3]. The distribution of CRP2 within invadopodia and its intrinsic actin-bundling activity strongly suggest that it directs the assembly of the actin bundles running inside and along the whole length of invadopodia. Contrary to T-fimbrin which was reported to be dispensable for invadopodia formation and activity (as evaluated by the degree of matrix degradation) in breast cancer cells [9], CRP2 knockdown decreased the degradation index by about 50% (Figure 4D), a value remarkably similar to the one reported for fascin depletion [9]. Thus, in the context of breast cancer, CRP2 and fascin might have similar importance in invadopodia function. However, they are unlikely to function as fully redundant, exchangeable, actin bundling factors. They considerably differ in their domain organization and 3-D architecture, and in the mechanism by which they promote actin bundling. Fascin functions as a monomer with two majors actin-binding sites [48, 49]. In contrast, we recently established that CRP3, which shares the same overall domain organization with CRP2 and CRP1, dimerizes along the actin cytoskeleton of muscle cells, and that its C-terminal LIM domain functions as an actin-binding module whereas its N-terminal domain promotes dimerization [25]. Thus, CRP2 actin-bundling activity likely involves protein dimerization. Nuclear magnetic resonance-based studies have shown that the > 50 amino acid-long linker region connecting the two LIM domains of CRP2 is mostly unfolded and highly flexible, providing each LIM domain motional freedom and independent spatial orientation [50]. Such an overall conformational plasticity contrasts with the more compact folding of fascin consisting in two double β-trefoil domain lobes (each containing one actin-binding site) hold together by a polar interface [13, 51]. The structural specificities of CRP2 and fascin likely account for their respective selectivity for AF polarity. Indeed, whereas CRP2 can crosslink AFs in both parallel and antiparallel orientations (Figure 5), fascin, whose folding restricts movements between its actin-binding domains, exclusively assembles tightly packed unipolar bundles [25, 49]. Although direct evidence is lacking, invadopodia are thought to contain unipolar bundles with their fast growing, barbed, ends oriented towards the tip. In this context, the structural flexibility of CRP2 and its low selectivity for AF polarity likely allow it to cooperate with fascin to assemble/stabilize unipolar bundles in breast cancer cell invadopodia. Nevertheless, the existence of a mixed polarity bundle population cannot be ruled out.

How is CRP2 expression regulated in breast cancer cell remains to be explored. However, the striking association between CRP2 up-regulation and the mesenchymal phenotype in breast cancer cell lines suggests that CRP2 is part of the EMT process whose role in tumor cell invasion and metastasis is increasingly recognized [52]. Interestingly, in vascular smooth muscle cells, CRP2 expression is driven by TGF-β [53], a potent inducer of EMT in cancer cells [54]. In this context it is worth mentioning that Hic-5, a four LIM domain-containing protein, was shown to be a key mediator of TGF-β-induced EMT, invadopodia formation and cell invasion [35]. Noticeably, ectopic expression of Hic-5 in epithelial, non-invasive, breast MCF-10A cells is sufficient to promote invadopodia formation and ECM degradation, suggesting that Hic-5 can recruit other key invadopodia components, and/or activate signaling pathways driving invadopodia formation. Remarkably, in fibroblasts, CRP2 and Hic-5 physically interact and co-regulate cell contractibility in response to mechanical stress [55]. Thus, a seducing scenario that awaits further examination is that Hic-5 and CRP2 also interact at invadopodia and contribute to invadopodia response to mechanical cues. Although Hic-5 localizes to the outer ring of invadopodia [35] whereas CRP2 localizes at the actin core, Hic-5 might recruit CRP2 during the early phase of invadopodia formation, following which CRP2 would translocate to and bundle nascent AFs. TGF-β, which was also reported to induce fascin expression in breast cancer cells, would thus coordinate up-regulation of key cytoskeletal proteins for invadopodia formation.

Targeting invadopodia represents an attractive alternative to MMP inhibitor-based strategies, which so far failed in clinical trials due to the lack of inhibitor specificity and unacceptable sides effects [56]. In this context, CRP2 emerges as a new potential therapeutic target to treat metastatic breast cancers. Importantly, mice lacking CRP2 are viable, fertile and only exhibit subtle alteration of cardiac ultrastructure [57]. It is thus conceivable that targeting CRP2 in patients would only cause minor side effects. Recently, small molecules impairing fascin bundling activity were shown to inhibit metastasis in breast cancer models with no toxicity in mice [13, 14], highlighting that the actin cytoskeleton, and more particularly actin bundlers, are realistic targets for anti-metastasis therapy. Our results call for the development of optimized strategies aimed at co-targeting fascin and CRP2. In support of this idea, the depletion of either fascin or CRP2 is not sufficient to fully abolish invadopodia-mediated ECM degradation *in vitro* ([9] and Figure 4, respectively). CRP2-based therapy might also turn out being an alternative to anti-fascin molecules in case they fail in clinic trials or to overcome resistance issues.

## MATERIALS AND METHODS

### Cell lines and cell culture

MCF-7 and MDA-MB-231-luc-D3H2LN (MDA-MB-231 luc) cells were purchased from ATCC and Perkin Elmer, respectively. The other cell lines used in this study including, MDA-MB-231, T47D, BT474 and SKBR3 cells were collected from cell line collections available at the Luxembourg Institute of Health or University of Luxembourg (kind gifts from Elisabeth Reckinger-Schaffner, Life Sciences Research Unit). MCF-7-derived 1001 and SNAIL 6SA cells were kindly provided by Dr. Bassam Janji (Luxembourg Institute of Health and University) with the permission of the respective inventors (see Acknowledgements). MCF-7-snail 6SA were maintained in DMEM:F12 (1:1; Lonza) supplemented with 1mM Sodium Pyruvate (Lonza) and 500 μg/ml G418 (Sigma-Aldrich). MCF-7, MDA-MB-231, MDA-MB-231-luc and 1001 cells were maintained in DMEM high glucose with L-glutamine medium (Lonza). The other cells were cultured in complete growth medium following ATCC recommendations. All the media were supplemented with 10% (v/v) fetal bovine serum (FBS, Life Technologies), 100 U/ml penicillin and 0.1 mg/ml streptomycin (Sigma-Aldrich). Cells were grown in a humidified atmosphere at 5% CO_2_ and 37°C. For MMP-9 induction and gelatinase zymography analyses, cells were incubated in serum-free medium and treated with PMA (100 ng/ml, Sigma-Aldrich) for 24 h. The conditioned media from PMA-treated and control cells were centrifuged at 4°C for 10 min at 300 × *g* and the supernatants were collected for subsequent analyses.

### CRP2 knockdown and ectopic expression

CRP2 knockdown was achieved by two pGIPZ lentiviral shRNAs (clone ID: V3HS_327411 and V3HS_327407, designated as shCRP2a and b). A non-silencing shRNAs (RHS4346, sh-; GE Dharmacon) was used as a control. For rescue experiments, the shCRP2a recognition site in the coding sequence of CRP2 was mutated (silent mutations) by overlapping PCR and the resulting CRP2mut_shCRP2a fragment was inserted into pcDNA3.1 (−) HA *via* BamHI an XhoI. The CRP2mut_shCRP2a-HA fragment was then PCR amplified and inserted into pCDH-EF1-MCS-IRES-Neo (hereafter referred to as pCDH, SBI, Systems Bioscience) *via* XbaI and NotI to generate the “shCRP2a/rescue” plasmid. For transwell, MMP-9 zymography, chemotaxis and MMP-9-expression analyses, CRP2 knockdown was achieved by transfecting MDA-MB-231-luc or Hs578T cells with CRP2 specific siRNAs (siCRP2; target sequence: 5′-TTGGATTTGTTGTAGGCCTGT-3′; Eurogentec) using DharmaFECT^TM^ transfection reagent (GE Dharmacon). A non-targeting siRNA (siCtr; Eurogentec) was used as a control. Transfected cells were kept 48 h in culture prior subsequent analyses, and CRP2 knockdown was confirmed by western blot. For stable expression of CRP2-GFP and GFP (control) under control of the CMV promoter in MDA-MB-231-luc cells, the insert present in the lentiviral expression plasmid “L134” (kindly provided by Dr. Birke Bartosch; INSERM, Lyon, France) was replaced with eGFP amplified from the pEGFP-N1 expression vector (Clontech). The CRP2 coding sequence (clone ID: BC000992) was subsequently inserted in N-terminal of eGFP *via* XhoI and BamHI restriction sites to generate the “L134_CRP2-GFP” expression plasmid. For stable expression of CRP2-HA in MCF7 cells, CRP2-HA fragment was inserted into empty pCDH vector *via* XhoI and BamHI restriction sites to generate the “pCDH-CRP2-HA” expression plasmid subsequently used for MCF7 cell lentiviral transduction. To produce the glutathione *S*-transferase (GST)-fused CRP2 recombinant protein, the coding sequence of CRP2 was subcloned into the bacterial expression vector pGEX-4T1 (GE Healthcare). GST-fused CRP2 was expressed in BL21 bacteria and purified using glutathione-agarose resin according to the manufacturer's instructions (Themo Scientific Pierce). Subsequently, GST was removed by thrombin cleavage. Lentivirus production was achieved by co-transfecting lentiviral pGIPZ shRNAs or expression plasmids with packaging and envelope plasmids in HEK293T cells using Xtreme transfection reagent (Roche). MDA-MB-231-luc-D3H2LN or MCF-7 cells (pCDH-CRP2-HA) were infected with virus, and transduced cells were selected with 0.5 μg/ml puromycin (Sigma-Aldrich; shCRP2a, b, sh-, L134_CRP2-GFP) or 1 mg/ml G418 (Sigma-Aldrich; shCRP2a/rescue; pCDH-CRP2-HA).

### RNA extraction and RT-qPCR

Total RNA was extracted with RNeasy Mini Kit (Qiagen) according to the manufacturer's instructions. Purified RNA was reverse-transcribed to cDNA using the SuperScript III cDNA synthesis kit (Invitrogen). qPCR reactions with specific primers for *MMP-9* (Qiagen), CRP1 (F:GAAGAGGTTCAGTGCGAAGG; R:CCAGATTCTTCTTGCAGACCA, Eurogentec), CRP3 (F: CTCGATGTGGCAAGTCAGTC; R:AGATGGCACAGCGGAAAC, Eurogentec) and *GAPDH* (F: AGCCACATCGCTCAGACAC; R: GCCCAATACGACCAAATCC, Eurogentec) were performed using SYBR Green I (Qiagen) in a Applied Biosystems ViiA^TM^ 7 Real-Time PCR System (Life Technologies). The 2^−ΔΔCt^ method was used to determine relative gene transcript levels normalized to the *GAPDH* reference gene.

### Protein extraction and western blot

Extraction of total protein from cell lysates was prepared in RIPA lysis buffer (Millipore) supplemented with protease and phosphatase inhibitor mixture (Roche). Twenty micrograms of proteins were separated by SDS-PAGE and transferred to a PVDF membrane (Millipore). Primary antibodies against the following proteins were used: CRP1 (079K2866, Sigma-Aldrich), CRP2 (HPA045617, Sigma-Aldrich), CRP3 (sc-166930, Santa Cruz Biotechnology), MMP-9 (#13667, Cell Signaling Technology), GADPH (#5174, Cell Signaling Technology) and tubulin (T5168, Sigma Aldrich). As secondary antibodies we used HRP-conjugated goat anti-rabbit IgG (111-035-003, Jackson ImmunoResearch) and HRP-conjugated goat anti-mouse IgG (A4416, Sigma-Aldrich). Protein bands were detected using the SuperSignal™ West Femto Chemiluminescent Substrate (Thermo Scientific) and visualized by ImageQuant LAS 4000 (GE Healthcare Life Science). Protein levels were quantified using ImageJ (NIH, Bethesda, USA).

### Cell proliferation assays

For MTT-based colorimetric assay, 10^3^ cells per well were seeded in 96-well plates and their growth was monitored over 6 days. Briefly, cells were incubated for 4 h in 0.5 mg/ml Thiazolyl Blue Tetrazolium Bromide (Sigma-Aldrich) and lysed in dimethyl sulfoxide (DMSO). The optical density was measured at 540 nm with FLUOstar OPTIMA (BMG LABTECH). For [^3^H]Thymidine incorporation-based assay, 3 × 10^3^ cells per well in 96-well plates were starved in serum-free DMEM medium for 24 h, and subsequently stimulated for proliferation in complete medium for 18h. Cells were then labeled with 0.25 μCi [^3^H]thymidine for 6 h and harvested onto UniFilter-96 GF/C plates using a Unifilter-96 Harvester (Perkin-Elmer). MicroScint-20 liquid scintillation cocktail was added to the dried filter plates at room temperature. The amount of incorporated tritium was measured using TopCount NXT^TM^ (Perkin-Elmer) Microplate Scintillation and Luminescence Counter (Perkin Elmer). For 3D spheroid proliferation assay, spheroids were prepared in 96-well plates coated with 1.5% Noble agar (BD Difco) prepared in serum-free DMEM medium. One thousand cells per well were centrifuged at 500 × *g* and incubated for 3 h at 37°C and 5% CO_2_. An ECM gel was prepared from Engelbreth-Holm-Swarm murine sarcoma (EHS matrix, Sigma-Aldrich) diluted to 1 mg/ml in complete DMEM growth medium, and added to clustered cells to induce spheroid formation. Fresh medium was added on the top of spheroids and changed every three days. Spheroids growth was monitored during 6 days. Spheroid diameter was measured in ImageJ (NIH, Bethesda, USA), and an average spheroid volume was calculated from 25 spheroids for each cell line.

### Cell migration and invasion assays

#### Scratch-wound migration assays

96-well plates were coated with rat tail collagen I (200 μg/ml, ibidi) in PBS and 2 mM acetic acid overnight at 37°C. After washing, 4 × 10^4^ cells per well (shCRP2a and b, and sh-) were seeded to form a confluent monolayer for 4 h. Homogeneous scratch wounds were generated by using a 96-well tool WoundMaker^TM^ (Essen BioScience). The cell free area was imaged every 3 h until the cells from both sides merged. Wound confluence was measured at each time point using the automated image acquisition and processing system IncuCyte (Essen BioScience). For scratch-wound invasion assays, cells and wounds were prepared as above described. After wounding, cells were embedded in a 3D collagen I gel (2 mg/ml, Ibidi) following manufacturer's instructions and the gap closure was measured after 72 h. Prior to chemotaxis assays, MDA-MB-231 and Hs578T cells were starved 24 h in serum-free DMEM medium without or with 1% FBS, respectively. After starvation, MDA-MB-231 cells (1 × 10^5^) and Hs578T cells (0.5 × 10^5^) were embedded in rat tail collagen I (Ibidi) gel (2 mg/ml and 1.5 mg/ml, respectively), and loaded in a μ-Slide Chemotaxis ^3D^ slide (Ibidi) and incubated at 37°C for 1 h to induce collagen gel polymerization. After polymerization, the two chambers flanking the channel were filled with DMEM medium supplemented or not with 10% FBS (and EGF 50 ng/ml in case of Hs578T cells), respectively. For each condition, about 150 cells (3 × 50) were tracked over 2 days using the live cell imaging Cell-IQ^TM^ system (CM Technologies; 1 picture every 20 minutes). Single cell tracking was performed in ImageJ with MTrackJ pluging (NIH, Bethesda, USA), and average velocities (μm/min^−1^) were calculated. MDA-MB-231 cell 2D migration velocity was determined as above described except that cells were loaded on a collagen IV coated μ-Slide Chemotaxis^2D^ slide (Ibidi).

For transwell analyses, 24-well inserts (Greigner) were filled with 100 μl of EHS matrix (330 μg/ml in DMEM medium) and incubated 2 h at 37°C. MDA-MB-231 cells (5 × 10^4^), Hs578T cells (3 × 10^4^), and MCF-7 cells (5 × 10^4^) were loaded on the insert in DMEM without serum (MDA-MD-231-luc cells) or supplemented with 1% FBS (HS578T cells) or 2% FBS (MCF-7 cells). The lower well was filled with DMEM supplemented with 10% FBS (MDA-MB-231 cells), or 10% FBS and 50 ng/ml EGF (HS578T and MCF-7 cells) as chemoattractant. The total number of cells and the number of cells that had invaded after 24 h (48 h in case of MCF-7 cells) were evaluated by MTT, and the percentage of invasion was calculated and normalized to siCtr cells (set to 100%).

### Gelatinase zymography

Gelatinase zymography assays were performed in 10% (v/v) SDS-PAGE gel in the presence of 1 mg/ml gelatin from bovine skin type B (Sigma-Aldrich). Conditioned media (20 μL/sample) from PMA-free or PMA-treated cells were prepared for electrophoresis under non-reducing conditions [46]. Gels were washed twice after electrophoresis in 2.5% (v/v) Triton X-100 for 30 min and incubated in a developing buffer containing 100 mM Tris-HCl, 5 mM CaCl_2_ and 0.005% (v/v) Brij-35 (pH 8.0) (Sigma-Aldrich) overnight at 37°C. Gels were stained for 30 min in a staining buffer containing 0.5% Coomassie Brilliant Blue R-250 in 50% (v/v) methanol and 10% (v/v) acetic acid (Sigma-Aldrich).

### Confocal microscopy and immuno-fluorescence

Cells were fixed in 4% paraformaldehyde (PFA, Thermo Scientific), permeabilized with 0.1% (v/v) Tween 20, incubated in the blocking buffer (5% Bovine Serum Albumin, BSA and 5% Normal Goat Serum, NGS, Sigma-Aldrich) for 1 h and incubated with primary antibodies overnight at 4°C. Cells were incubated with appropriate secondary antibodies for 4 h at room temperature. Analyses were performed in mounting medium (Citifluor AF1, Agar Scientific) using a Zeiss LSM510 Meta laser scanning confocal microscope equipped with a ×63/1.4-numerical-aperture (NA) oil immersion Plan Apochromat objective. Stacks with 0.4 μm optical sections were captured and processed for deconvolution by using Huygens essential Software (SVI, Netherlands) to enhance the signal-to-noise ratio. To investigate CRP2 localization in invadopodia, cells were cultured on Cy3-conjugated gelatin-coated coverslips (QCM^TM^ Gelatin Invadopodia Assay, Millipore) for 6 h and CRP2-GFP was co-localized with cortactin (antibody clone 4F11, Millipore) and actin (Acti-stain^TM^ 670 phalloidin, Cytoskeleton). To localize CRP2 in elongated invadopodia, cells were grown on EHS matrix-coated ThinCert^TM^ Tissue Culture Inserts (Greiner) with 1-μm-diameter pores [9]. Invadopodia elongation was stimulated with conditioned culture medium containing 15% FBS and 50 ng/ml EGF (Preprotech) for 24 h. For localization experiments in invading cells, we used a circular invasion assay similar as the one described in [37]. Briefly, 5 × 10^5^ cells were plated around a silicone culture-insert (ibidi) in a round μ-dish (35mm, ibidi) to create a large cell free region. After 24 h, the insert was removed and cells were overlaid with 4.5 mg/ml EHS matrix. EHS matrix was polymerized during 4 h at 37°C prior addition of culture medium. After 48 h cells were fixed and CRP2-GFP was co-localized with cortactin and actin. In addition to CRP2-GFP detection, endogenous CRP2 was immunolocalized using the same antibodies as in western blot.

The skewness analysis for actin bundling quantification was performed as previously described [25]. In brief, regions of interest (ROI, 13 × 13 μm) containing stress fibers labeled with Acti-stain 555 phalloidin were acquired using optimized and identical settings. The skewness of signal intensity distribution of AF pixels was calculated using the software plug-in Kbi_Filter2d (ThinLine), developed by Higaki and coworkers [58] (available at http://hasezawa.ib.k.u-tokyo.ac.jp/zp/Kbi/HigStomata). Skewness average values were calculated from three independent experiments, including 200 ROI, for each condition.

### Fluorescent gelatin degradation assay

5 × 10^4^ cells were seeded on a 16 mm coverslip coated with a 6:1 mix of unlabeled:Cy3-labelled gelatin according to the manufacturer's instructions (QCM^TM^ Gelatin Invadopodia Assay; Millipore). After 18 h at 37°C, cells were fixed and stained for actin (Acti-stain^TM^ 670 phalloidin, Cytoskeleton). ECM degrading cells were defined as cells with an underlying black area depleted of fluorescent gelatin. Active cells were scored and expressed as percentage of the total cell population. In addition, the degraded area per cell was measured using the threshold tool of ImageJ software, and a degradation index corresponding to the ratio of degraded gelatin surface per cell was calculated.

### Total internal reflection fluorescence (TIRF) microscopy

TIRF microscopy observations were performed as previously described in [25]. A 3:1 mix of cold (Cytoskeleton) and Alexa Fluor 488-labeled actin (1 μM; Invitrogen) was induced to polymerize in NEM-myosin-coated perfusion chambers as shown in [59]. The chamber was perfused with actin alone or in combination with 3 μM recombinant CRP2. Actin filaments were imaged by TIRF microscopy on a Zeiss Axiovert 200M inverted microscope equipped with a ×100/1.46-NA Alpha Plan-Apochromat TIRF objective. Time lapse images were acquired at 5 s intervals over 45min with a Zeiss Axiocam HR camera. Images were analyzed using ImageJ. Kymograph was built along actin bundles with the MultiKymograph plugin (http://www.embl.de/eamnet/html/body_kymograph.html).

### Immunohistochemistry

A triple negative breast cancer tissue array (BRC964) was purchased from Pantomics, Inc. (Richmond, CA, USA). Immunohistochemical staining was performed using rabbit anti-human CSRP2 (HPA045617, Sigma-Aldrich) after an optimal dilution (1/250) was determined using a universal TMA UNC241 (Richmond, CA, USA). Rabbit anti-cytokeratin and normal rabbit serum were used as positive and negative controls. Heat-induced epitope retrieval was performed using a pressure cooker and Tris-EDTA buffer. Polymer-based detection system was used for detection according to Pantomics' standard protocol.

### Experimental and spontaneous metastasis breast cancer models

Animal work was conducted in accordance to the national and international regulations, and with protocols approved by the Ministry of Agriculture and Ministry of Health. Highly invasive MDA-MB-231-luc-cells expressing an shRNA targeting CRP2 (shCRP2a) or a control shRNA (sh-) were trypsinized, washed in PBS twice and filtered onto a 50 μm filter (Partec CellTrics). In the experimental metastasis model, 10^6^ cells in 0.1 ml PBS were injected into the lateral tail vein of 6-week-old female athymic nu/nu mice (Charles River). Cell implantation to the lungs was immediately controlled by bioluminescence imaging (IVIS imaging system). After 5 weeks, mice were sacrificed by cervical dislocation after isoflurane (2%) anesthesia. Lungs were collected and the number of GFP (transduction marker) positive metastatic lesions was analyzed under a fluorescent stereomicroscope and using ImageJ (NIH, Bethesda, USA). The other organs and tissues of mice from the two groups were also carefully checked for the presence of metastases. In the spontaneous metastasis model 5 × 10^5^ cells in 50 μL EHS matrix diluted 1:1 in PBS were injected into one mammary fat pad of 6-week-old NOD.Cg-*Prkdc*^scid^ *Il2rg^tm1Wjl^*/SzJ (NSG) mice (Charles River). Primary tumor dimensions (length, width and height) were periodically measured using calipers and the tumor volume was calculated according to the formula: ½ × L × W × H. After 20 days, mice were sacrificed by cervical dislocation and lungs were collected and the number of GFP (transduction marker) positive metastatic lesions was analyzed as above described.

### Statistics

All numerical data are shown as mean ± SE. Error bars represent standard error. Kaplan-Meier survival plots [29], hazard ratio with 95% confidence intervals and Logrank P values were used to test for associations between CRP2 expression (Affy probe 207030­_s_at), and metastasis-free survival in breast cancer patients. Statistical significance was determined by Mann-Whitney U test for the *in vivo* lung metastasis models, Z-test for two population proportions for the gelatin degradation assays (active cells), and unpaired two-tailed Student's *t* test for the other analyses. *P* values < 0.05 were considered statistically significant.

## SUPPLEMENTARY MATERIAL FIGURES AND MOVIES








