# Supplementary material for: CRP2, a new invadopodia actin bundling factor critically promotes breast cancer cell invasion and metastasis
Source: Oncotarget. 2016 Feb 11;7(12):13688–705. doi: 10.18632/oncotarget.7327 (PMC4924671; doi:10.18632/oncotarget.7327)
Supplement: Supplementary file 1 [file oncotarget-07-13688-s001.pdf]

CRP2, a new invadopodia actin bundling factor critically promotes breast cancer cell invasion and metastasis

Supplementary Material

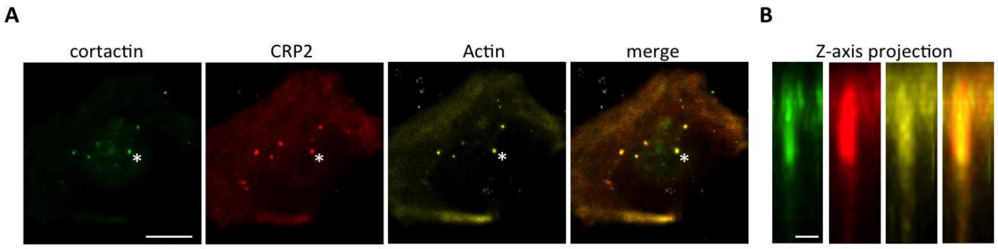

Supplementary Figure 1. Endogenous CRP2 accumulates in invadopodia. MDA-MB-231-luc cells were grown on EHS matrix-coated transwell with 1-µm-diameter pores. Invadopodia elongation was stimulated with 15% FBS and 50 ng/ml EGF. After 24 h, both endogenous CRP2 and cortactin were immuno-labeled and the actin cytoskeleton was stained with Acti-stain 670 phalloidin. A. Focal plane near the ventral cell surface. B. Z-axis projection of the invadopodia labeled by an asterkis in (A). Bars: 10 µm (A) and 1 µm (B).

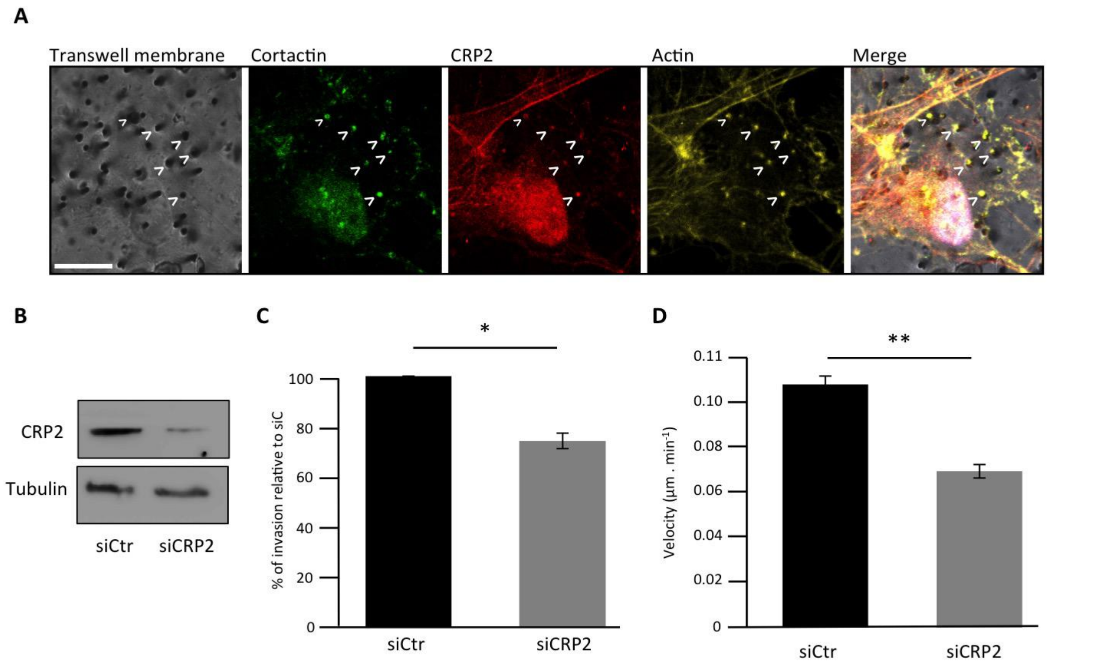

Supplementary Figure 2. CRP2 contributes to cell invasion and localizes to mature invadopodia in Hs578T breast cancer cells. A. Endogenous CRP2 (red) colocalizes with cortactin (green) and actin (yellow) in Hs578T cell invadopodia which have extended through a 1-µm-diameter pore transwell membrane. Arrowheads show labeled invadopodia. B. CRP2 protein level in Hs578T cells transfected with a control siRNA (siCtr) or a siRNA targeting CRP2 transcripts (siCRP2). C. Transwell assay performed with control and CRP2-depleted Hs578T cells. Invading cells at 24 h were quantified by MTT assay, and the results were normalized to siCtr cells (set to 100%). D. Velocity of control and CRP2-depleted Hs578T cells invading a collagen I 3 D gel. For each condition, 150 (3 x 50) cells were tracked over 48 h and an average velocity was calculated. Error bars denote standard error. Significant levels: **: p<0.00001, *: p<0.005. Bars = 300 µm (B) and 150 µm (D and F). Bar = 20 µm.

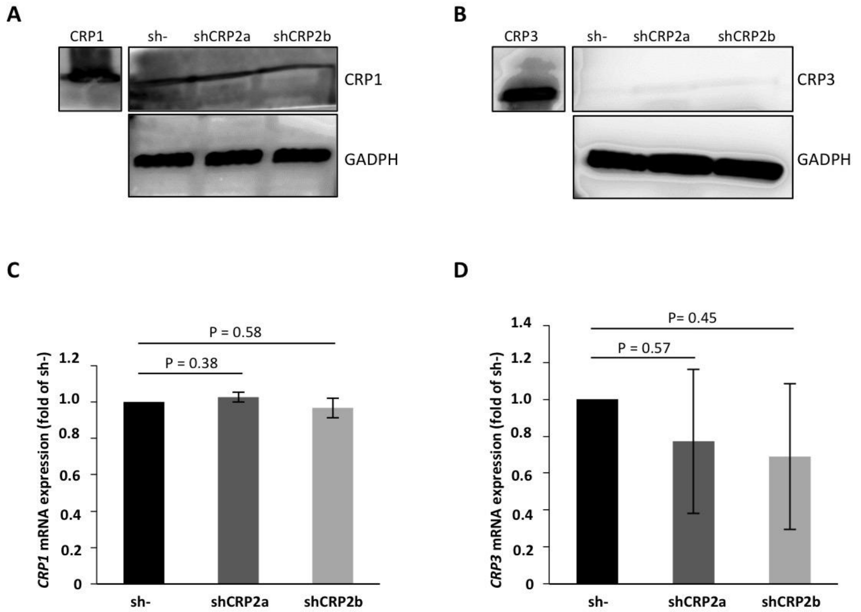

Supplementary Figure 3. CRP1 and CRP3 expression at protein and mRNA levels in CRP2-depleted (shCRP2a and shCRP2b) and control (sh-) MDA-MB-231-luc cell lines. A and B. Western blot analyses for CRP1 (A) and CRP3 (B) protein expression. One microgram of recombinant CRP1 and CRP3 proteins (available in our laboratory) were used as respective positive controls (left panels) and GADPH was used as a loading control. C and D. Real-time qPCR analyses for CRP1 (C) and CRP3 (D) mRNA expression. The data originate from 3 independent experiments and were normalized to CRP1 or CRP3 expression in control sh- cells (set to 1). Error bars denote standard error. Significant levels: p values are given in the charts.

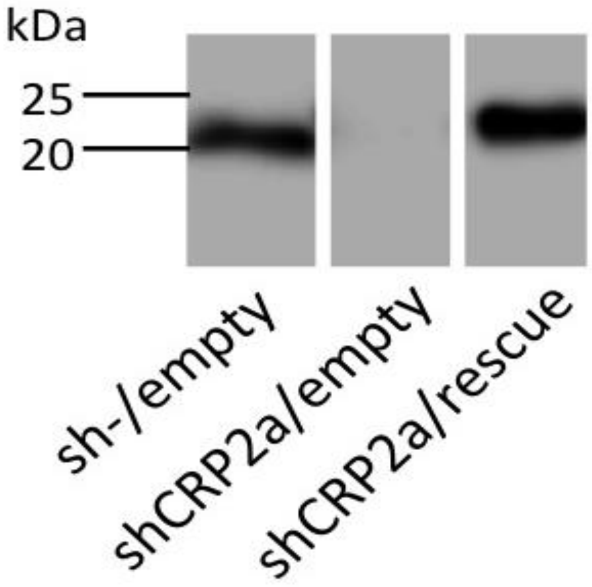

Supplementary Figure 4. Rescue of CRP2 expression in shCRP2a cells. The shCRP2a recognition site in CRP2 cDNA was mutated (silent mutation). The mutated cDNA ("rescue") was fused to a HA tag coding sequence and introduced in shCRP2a cells by lentiviral transduction. As controls, an empty vector ("empty") was introduced in sh- and shCRP2 cell lines. Total protein extract from each cell line was probed with anti-CRP2 antibodies. The slight increase in molecular weight of CRP2 in shCRP2a/rescue cells (right panel) was due to the HA tag.

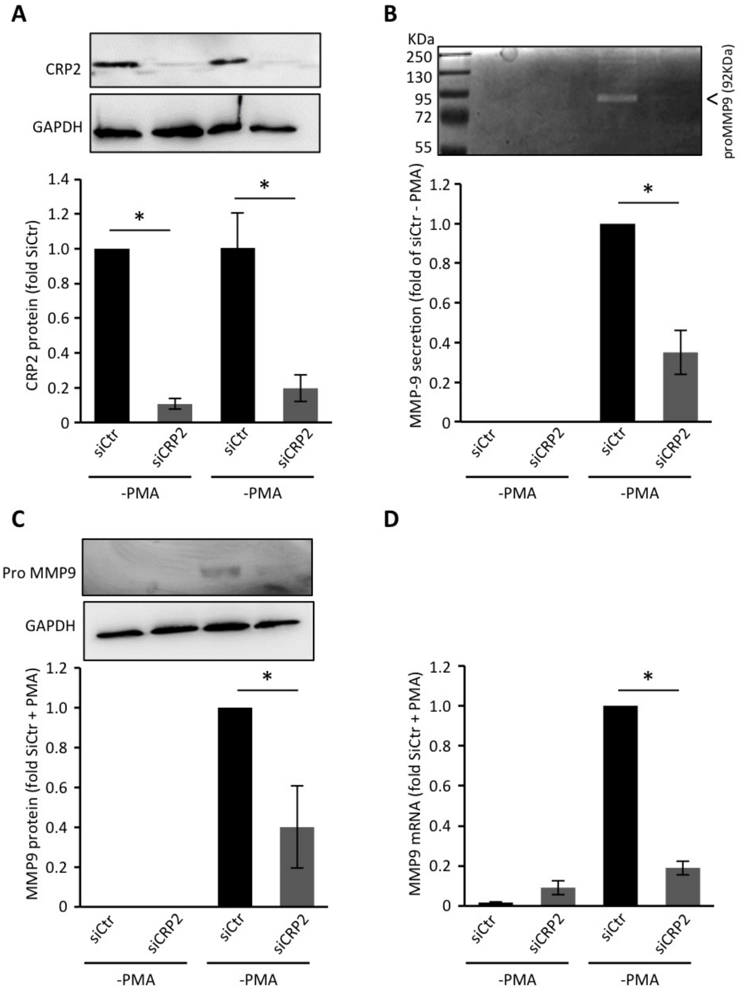

Supplementary Figure 5. CRP2 regulates MMP9 expression in Hs578T cells. A. CRP2 protein level in Hs578T cells transfected with a control siRNA (siCtr) or a CRP2 targeting siRNA (siCRP2). Cells were cultured 48 h in serum-depleted DMEM and subsequently treated with or without PMA (100 ng/ml) for an additional 24 h. Average expression values (lower panel) were expressed as fold of CRP2 protein in untreated (-PMA) control siCtr cells which was set to 1. B. Gelatin zymography assays. The medium of the cell cultures described in (A) was collected and assessed for its content in secreted pro-MMP-9. The results were expressed as fold of pro-MMP-9 secretion in PMA-treated control siCtr cells which was set to 1. C and D. Expression levels of cytosolic MMP-9 protein (C) and MMP-9 mRNA (D) in the cell cultures described in (A) as assessed by western blot and real-time qPCR analyses, respectively. The results were expressed as fold of pro-MMP-9 expression in PMA-treated control siCtr cells which was set to 1. The data originate from at least 3 independent experiments. Error bars denote standard error. Significant levels: *: p<0.01.

Supplementary Movies.

Supplementary Movie 1. Successive focal planes showing CRP2-GFP and actin (Acti-stain 670 phalloidin) localization in elongated invadopodia (from the ventral cell surface to the tip of invadopodia). See also Figure 2 F-I.

Supplementary Movie 2. Formation and elongation of CRP2-induced actin bundles. See also Figure 3E.

Supplementary Movie 3. Growth of CRP2-induced actin bundles. See also Supplementary Figure 3F.
